# Evaluating GPT-4 Responses on Scars or Keloids for Patient Education: Large Language Model Evaluation Study

**DOI:** 10.2196/78838

**Published:** 2026-02-27

**Authors:** Mingjun Rao, Tang Xiujun, Wang Haoyu

**Affiliations:** 1 Department of Plastic Surgery, Guizhou Provincial People's Hospital, 83 Zhongshan East Road, Nanming District, Guiyang, 550002, China, 86 15343315902

**Keywords:** scar, keloid, GPT-4, patient education, generative AI, generative artificial intelligence, readability, large language model

## Abstract

**Background:**

Scars and keloids impose significant physical and psychological burdens on patients, often leading to functional limitations, cosmetic concerns, and mental health issues such as anxiety or depression. Patients increasingly turn to online platforms for information; however, existing web-based resources on scars and keloids are frequently unreliable, fragmented, or difficult to understand. Large language models such as GPT-4 show promise for delivering medical information, but their accuracy, readability, and potential to generate hallucinated content require validation for patient education applications.

**Objective:**

This study aimed to systematically evaluate GPT-4’s performance in providing patient education on scars and keloids, focusing on its accuracy, reliability, readability, and reference quality.

**Methods:**

This study involved collecting 354 questions from Reddit communities (r/Keloids, r/SCAR, and r/PlasticSurgery), covering topics including treatment options, pre- and postoperative care, and psychological impacts. Each question was input into GPT-4 in independent sessions to mimic real-world patient interactions. Responses were evaluated using multiple tools: the Patient Education Materials Assessment Tool-Artificial Intelligence for understandability and actionability, DISCERN-AI for treatment information quality, the Global Quality Scale for overall information quality, and standard readability metrics (Flesch Reading Ease score, and Gunning Fog Index). Three plastic surgeons used the Natural Language Assessment Tool for Artificial Intelligence to rate the accuracy, safety, and clinical appropriateness, while the Reference Evaluation for Artificial Intelligence tool validated references for reference hallucination, relevance, and source quality. We conducted the same analysis to assess the quality of GPT-4–generated content in response to questions from 3 medical websites.

**Results:**

GPT-4 demonstrated high accuracy and reliability. The Patient Education Materials Assessment Tool-Artificial Intelligence showed 75.5% understandability, DISCERN-AI rated responses as “good” (26.3/35), and the Global Quality Scale score was 4.28 of 5. Surgeons’ evaluations averaged 3.94 to 4.43 out of 5 across dimensions (accuracy 3.9, SD 0.7; safety 4.3, SD 0.8; clinical appropriateness 4.4, SD 0.5; actionability 4.1, SD 0.8; and effectiveness 4.1, SD 0.8). Readability analyses indicated moderate complexity (Flesch Reading Ease Score: 50.13; Gunning Fog Index: 12.68), corresponding to a 12th-grade reading level. Reference Evaluation for Artificial Intelligence identified 11.8% (383/3250) hallucinated references, while 88.2% (2867/3250) of references were real, with 95.1% (2724/2867) from authoritative sources (eg, government guidelines and the literature). The overall results about questions from medical websites were consistent with the answers to Reddit questions.

**Conclusions:**

GPT-4 has serious potential as a patient education tool for scars and keloids, offering reliable and accurate information. However, improvements in readability (to align with sixth to eighth grade standards) and reduction of reference hallucinations are essential to enhance accessibility and trustworthiness. Future large language model optimizations should prioritize simplifying medical language and strengthening reference validation mechanisms to maximize clinical utility.

## Introduction

Scars and keloids are common skin healing outcomes [1], often causing discomfort during the proliferative phase [2]. When located on visible areas such as the face, they can severely impact a patient’s appearance, leading to psychological distress such as low self-esteem, anxiety, and depression, which may further hinder social interactions and career development [3]. Scars near joints may cause functional limitations, while perineal scars can result in long-term complications such as dyspareunia and dysmenorrhea [4,5]. Many patients urgently seek to improve both the aesthetic and functional aspects of scars or keloids. However, treatments often require multimodal approaches over weeks to months, making high patient adherence crucial. Consequently, access to accurate, timely, and comprehensive management information is critical for patients to better understand treatment expectations, options, prognosis, and potential complications [6-9].

Currently, patients increasingly rely on internet-based health information [10]. This trend faces multifaceted challenges, including information overload, variability in source credibility and content accuracy, and the health literacy required to understand the contents [11,12]. Notably, many online resources on scars or keloids are often unreliable, fragmented, or difficult to understand, failing to tackle the fundamental needs of patients with scars or keloids [13].

In recent years, artificial intelligence (AI) tools powered by large language models (LLMs), such as GPT-4 (used by ChatGPT), have demonstrated significant potential in delivering medical information [14]. GPT-4’s capacity to generate natural language responses through interactive conversions could aid users in understanding intricate medical concepts, treatment, and management strategies, positioning it as a potentially valuable alternative to traditional search engines for accessing knowledge associated with scars or keloids [15-17].

It is reported that 52% of US adults have used LLMs, and GPT-4, as a leading LLM, receives over 5 billion monthly visits. In total, 39% of LLM users have used LLMs for health care queries [18]. Despite the increasing use of LLMs in health care [19-21], there remains a research gap, and it is currently unclear whether GPT-4 can generate high-quality patient education content related to scars and keloids. Thus, we conducted this study to comprehensively assess the use of GPT-4 in keloid and scar patient education by performing a multidimensional evaluation (encompassing accuracy, reliability, readability, and reference quality) of GPT-4 responses.

## Methods

### Study Objective

This study aimed to investigate the potential of GPT-4 to provide reliable, accurate, readable, and actual medical information for patients with scars or keloids. To achieve this, we used GPT-4 (OpenAI) to evaluate its accuracy, reliability, readability, and hallucinations in answering questions related to treatments of scars or keloids.

### Question Collection

The research questions were manually collected by the authors from Reddit. First, the authors analyzed all posts on the “Hot” page (the most popular and recently active posts) of the r/Keloids subreddit, all posts on the r/SCAR subreddit, and all posts on the r/PlasticSurgery subreddit as of April 6, 2025. We extracted the main text of each post that included the keywords “Scar” or “Keloid” and organized the data using Microsoft Excel. Second, one author (MR) excluded the posts that contained no questions, were duplicates, or had repeated questions. In addition, the same author (MR) performed the initial classification of the questions. To ensure reliability and minimize bias, another author (WH) independently reviewed the process. Consistency between the two authors’ classifications was confirmed through discussion. This data collection approach has been adopted in previous Reddit-based research [22]. Furthermore, we adopted 49 questions about keloids or scars from 3 medical websites.

### Ethical Considerations

The institutional review board of the People’s Hospital of Guizhou Province, affiliated with Guizhou University, deemed this study exempt from ethics approval.

### Quality Assessment

#### Overview

Each question was input individually into GPT-4. Following previous research protocols, a new chat session was initiated for each question to avoid context contamination and to simulate real-world patient interactions [23]. Consistent with real-world activities using GPT-4, no specialized prompt instructions were appended to the question inputs. The contents generated by ChatGPT-4 were evaluated using a modified version of existing health information quality assessment tools.

#### Patient Education Materials Assessment Tool-AI Tool

The Patient Education Materials Assessment Tool (PEMAT) was used to assess the understandability and actionability of ChatGPT-generated content. The original PEMAT includes 17 items for understandability and 7 for actionability. Since all contents generated by ChatGPT are pure text, the PEMAT was simplified to 8 items for understandability and 3 items for actionability (Multimedia Appendix 1). Each item received 1 point if it met the standard, and scores were reported as percentages. A score of 70% or greater was recorded as a “pass” per PEMAT guidelines [24].

#### DISCERN-AI Tool

The DISCERN standard, a previously validated tool to help health care consumers and professionals evaluate the quality of treatment information, was adapted for ChatGPT-generated content. Since all contents generated by ChatGPT are pure text, 7 items (questions 3‐9 from the 15-item DISCERN tool) were selected and scored on a 1 to 5 scale (Multimedia Appendix 2). Each output was rated as follows: very poor (7‐12 points), poor (13‐17 points), fair (18‐23 points), good (24‐28 points), and excellent (29‐35 points) [24,25].

#### Global Quality Scale

The Global Quality Scale (GQS) is a 5-point Likert scale used to evaluate information quality and the flow and ease of use of information. The scores range from 1 (low quality) to 5 (high quality), while scores of 4 or 5 indicated high-quality outputs, a score of 3 was considered moderate quality and scores of 1 or 2 were categorized as low quality.

#### Readability Assessment

The readability of the ChatGPT-generated content was evaluated using several established readability formulas, including Flesch Reading Ease score, Gunning Fog Index, Flesch-Kincaid Grade Level, Coleman-Liau Index, and Simple Measure of Gobbledygook (SMOG). Each output was copied into Microsoft Word and analyzed via the Readable website [26]. The Flesch Reading Ease score ranges from 0 to 100, and higher scores indicate greater readability. A score between 60 and 70 corresponds to reading levels of grades 8 and 9 and is generally understandable by the average adult. The Gunning Fog Index and Flesch-Kincaid Grade Level are used to estimate sentence complexity; the scores represent the years of formal education required to understand the contents. For example, a score of 12 implies the output is suitable for readers at the 12th-grade level. The Coleman-Liau Index is similar to the Gunning Fog Index and Flesch-Kincaid Grade Level but uses character counts instead of syllables, making it more suitable for languages where syllable counts may not accurately reflect complexity. The SMOG Index measures syllable density, often used to assess health information materials. A score of 12 indicates that the material is suitable for readers at the 12th-grade level or higher.

#### Natural Language Assessment Tool for Artificial Intelligence

Three experienced plastic surgeons independently reviewed each GPT-4–generated content using a specially developed Natural Language Assessment Tool for Artificial Intelligence (NLAT-AI) [24]. Using this tool, we assessed accuracy, safety, appropriateness, actionability, and effectiveness. Each output was rated using a 5-point Likert scale (1=strongly disagree, 5=strongly agree; Multimedia Appendix 3). All results were summarized using descriptive statistics.

#### Reference Evaluation for AI

Given known issues of LLM hallucination (ie, generating plausible but nonexistent references), a brief evaluation tool, Reference Evaluation for AI, was developed to analyze references provided in ChatGPT-generated content [27]. Each reference was verified through direct links or a Google search. The tool assessed (1) reference hallucination (whether references were real or fabricated), (2) relevance and consistency between references and AI output, and (3) source quality (based on the authority of the issuing institution or organization, such as government guidelines, health care organizations, or scientific research; Multimedia Appendix 4).

## Results

### Question Collection and Classification

A total of 507 posts were identified and analyzed (posts from the r/Keloids subreddit: n=193, 38.1%; posts from the r/Keloids subreddit: n=211, 41.6%; and posts from the r/Scar subreddit: n=103, 20.3%). After removing posts that merely shared information or were duplicates, 354 unique questions were obtained. The questions were categorized into 16 groups based on their contents (Table 1). Furthermore, we obtained 49 questions from 3 medical websites that included 38 unique questions (Table S1 in Multimedia Appendix 5).

**Table 1.** Questions on scars or keloids from Reddit (N=354).

Question group	Questions, n (%)
Questions on other respects	28 (7.9)
Questions on other treatments for scars or keloids	4 (1.1)
Questions on common treatments for scars or keloids	46 (13)
Questions on trauma-related scars or keloids	16 (4.5)
Questions on psychological issues caused by scars or keloids	9 (2.5)
Questions on at-home scar or keloid care	3 (0.8)
Questions on preoperative scar or keloid consultation	37 (10.5)
Questions on postoperative scar or keloid consultation	55 (15.5)
Questions on selection of treatments for scars or keloids	80 (22.6)
Questions on impact of scars or keloids on daily life	2 (0.6)
Questions on scar or keloid symptoms	7 (2)
Questions on scar camouflage	6 (1.7)
Questions on the impact of nutrition on scars or keloids	3 (0.8)
Questions on choosing physicians for scar or keloid treatment or related costs	32 (9)
Questions on old scars	14 (4)
Questions on scar or keloid prevention	12 (3.4)

### Evaluation of GPT-4–Generated Content

GPT-4 generated content that provided a wide range of medically accurate information. Using the PEMAT-AI, DISCERN-AI, and GQS patient education material evaluation tools, the output of GPT-4 was assessed, with all tools indicating high scores. The overall understandability score using PEMAT-AI easily surpassed the 70% threshold for acceptability (mean 75.5%, SD 12.2%). The DISCERN-AI tool resulted in an overall rating of “good” quality (mean 26.3, SD 3.4), with all 16 groups of questions rated as “good.” The GQS score averaged 4.3 out of 5 (SD 0.8), categorizing the outputs as high quality. More details are shown in Table S1 in Multimedia Appendix 6. Intraclass correlation coefficient (ICC) for PEMAT-AI, DISCERN-AI, and GQS were 0.73, 0.69, and 0.78, respectively (Table S2 in Multimedia Appendix 5). The results of the ICC demonstrated high reliability of the evaluation tools.

### Plastic Surgeons’ Evaluation via the NLAT-AI Tool

Using the NLAT-AI tool, 3 independent plastic surgeons evaluated the GPT-4–generated content. All dimensions of the contents received scores above the neutral midpoint of 3 on a 5-point Likert scale. The overall average scores for each dimension were as follows: accuracy 3.9 (SD 0.7), safety 4.3 (SD 0.8), appropriateness 4.4 (SD 0.5), actionability 4.1 (SD 0.8), and effectiveness 4.1 (SD 0.8). More detailed descriptive statistics for each question are presented in Table S2 in Multimedia Appendix 6. Internal validity tests showed an ICC of 0.76 (Table S2 in Multimedia Appendix 5), indicating high reliability.

### Readability Assessment

The results of the readability assessments indicated that the GPT-4–generated content was “difficult to read.” The average Flesch Reading Ease score was 50.1 (SD 8.1), which is considered moderately difficult. The Gunning Fog Index averaged 12.7 (SD 3.3), and the Flesch-Kincaid Grade Level was 12.4 (SD 2.5), indicating that the text was at a high school level (approximately suitable for individuals aged 16‐17 years). The Coleman-Liau Index averaged 12.8 (SD 2.6), and the SMOG Index averaged 11.3 (SD 3.16). More detailed evaluation results are shown in Table S3 in Multimedia Appendix 6.

### Reference Evaluation for AI Assessment

Most of the references provided in GPT-4’s output effectively supported the content. A total of 88.2% (2867/3250) of the references were from actual sources (actual websites or academic papers), while 383 hallucinated references were identified. Among these 2867 real references, 2746 (95.8%) references effectively supported the content. In addition, a total of 95.1% (2724/2867) of the real references were from authoritative sources (government guidelines, health care organizations, or scientific research). More detailed evaluation results are shown in Table S4 in Multimedia Appendix 6.

### The Assessment of Questions From Websites

The evaluation results of GPT-4 responses to website-sourced questions were broadly consistent with those from Reddit-derived questions across all assessments (Tables S3-S6 in Multimedia Appendix 1).

## Discussion

### Principal Findings

This is the first study to assess the overall quality of ChatGPT responses to real-world questions from Reddit about keloids or scars. The results revealed that the content generated by GPT-4 was generally comprehensive and aligned with current medical guidelines and the literature. Using several assessment tools, as well as plastic surgeons’ evaluations, the scores were robust, and the plastic surgeons’ evaluations were largely positive. The overall results indicate that GPT-4–generated content is reliable, accurate, safe, and actionable, despite there being room for improvement in terms of readability and hallucination.

Over 80% of dermatology outpatients obtain medical information through social media or the internet, with 47% considering it an important source of information [28]. Although patients have access to a wealth of information, studies evaluating the quality of online health information have identified significant deficiencies [29]. As for scars and keloids, the information available to patients contains a lot of low-quality content. A previous study assessing 88 websites related to “burn scars” showed that most of the commercial websites provided information of moderate to poor quality [13]. In contrast, LLMs provide a broad range of fundamentally accurate information and real-time dynamic interactions compared to traditional webpages [30,31]. As a leading LLM, GPT-4 exhibits certain advantages over other LLMs and has demonstrated top-tier performance across diverse evaluations in health care. In answering questions from the American Board of Surgery In-Training Examination, GPT-4 achieved an accuracy rate comparable to that of Copilot, while significantly outperforming Gemini [32]. In other fields of clinical medicine, GPT-4 also attained superior performance relative to other LLMs [33,34]. However, in a substantial number of evaluative scenarios, the performance of GPT-4 did not yield statistically significant differences when compared with Copilot or Gemini. Collectively, the performance of GPT-4 currently represents the best capability of LLMs.

In our study, experienced plastic surgeons evaluated the outputs of GPT-4, confirming that the contents were reliable and accurate. The accuracy of GPT-4 in patient education has also been studied in other clinical contexts (eg, rhinoplasty, sleep apnea, and prostate cancer) where it demonstrated high accuracy and strong reliability [24,35,36]. Such high accuracy and reliability suggest that LLMs such as GPT-4 can effectively address clinical questions from patients with scars or keloids, serving as a valuable auxiliary tool in clinical medicine.

Despite GPT-4’s significant potential in responding to keloid or scar patient queries, its outputs commonly had high reading difficulty. Our study revealed that the average reading level of GPT-4–generated content was at a high school level. The results suggest that ChatGPT does not always meet the comprehension needs of all patients. The relatively low readability of GPT-4 can hinder accessibility for certain socioeconomic populations with limited health literacy [37]. Among the latest generation of young adults in the United States, up to 13% have not graduated from high school. This rate reaches 20% among people of color (including African Americans and Native Americans) [38], who are also identified as high-risk groups for developing malignant scars [39]. Due to poor readability, GPT-4 has apparent barriers in its application among these populations [40,41]. To enhance the utility of LLMs for populations with lower educational attainment, it is recommended that developers consider training specialized LLMs based on datasets with good readability [42]. Biomedical text can be simplified through hyperparameter substitution techniques, improving patient understanding [43]. In addition, structured prompting can also contribute to enhancing readability [44].

Moreover, our study also revealed the existence of hallucination, where GPT-4 cited nonexistent references or websites. Fabricated references not only mislead readers and distort their understanding of keloid or scar but also—given the presence of numerous seemingly authoritative yet false information sources—may lead patients to overtrust the content generated by GPT-4 [45,46]. Given the presence of hallucinations, specific clinical diagnosis and treatment must rely on clinicians; LLMs can only serve as auxiliary tools. To address the hallucination issue in LLMs, it is recommended that developers effectively apply retrieval-augmented generation to retrieve documents from an external corpus (such as academic library systems), as this can significantly reduce the hallucinations [47-49]. Integrating external, structured knowledge sources (such as knowledge graphs, databases, or other domain-specific resources) into LLMs can also help ensure that LLMs produce responses with fewer hallucinations [50]. Furthermore, prompt engineering can mitigate hallucination by improving the reasoning capabilities [51].

GPT-4 can provide comprehensive and generally accurate information, which can further assist patients with keloids or scars in accessing timely and precise information. However, current LLMs exhibit limitations, such as hallucinations and relatively low readability; therefore, they are not recommended as the sole source of information for patients. Limited by the lack of clinical background in current LLMs; the insufficient ability to process audio, image, and video information; limited ability to access academic libraries; and the noninterpretability of black box algorithms, current LLMs still require further development to be adapted for applications in health care [52]. The AI agent, as a promising approach, can extend the capabilities of LLMs by enabling them to use external tools, plan and execute multistep tasks, as well as interact dynamically [53]. Multimodal LLM is promising to process text (eg, clinical notes and user-input questions), medical images (eg, photos and computed tomography scans), and videos (eg, treatment procedures) provided by patients, which will more effectively assist patients and health care providers in clinical practice about keloid and scar management [54].

### Limitations

Most of the questions collected from Reddit were posts from patients who had not yet sought medical care. Consequently, the questions posed may be biased toward pretreatment information needs, as fewer questions were reported during the treatment phase. This may compromise the generalizability of GPT-4’s evaluation across different patient care stages. In addition, Reddit users are concentrated in the age group of 18 to 49 years, with an average age of 23 years, and the majority are aged under 30 years. Thus, the data collected from Reddit clearly fails to represent the middle-aged and older population [55]. Relying solely on Reddit posts for data collection introduces demographic selection bias.

In terms of assessment tools, the qualitative assessment conducted by experienced plastic surgeons was inherently at risk of bias, given the surgeons’ attitudes toward the use of GPT-4. Nevertheless, they provided valuable insights owing to their in-depth understanding of scar and keloid education materials. Furthermore, exploratory assessment tools (DISCERN-AI, PEMAT-AI, and NLAT-AI) were used in this study, while their validity requires further testing. LLMs differ from traditional printed educational materials in that their responses to repeated queries of the same question are generated instantaneously and may vary. Currently, existing assessment tools lack the ability to detect such variability in LLM outputs when the same question is posed multiple times [56]. Furthermore, content generated by LLMs is often conveyed with excessive certainty, as these models lack the ability to accurately express information involving uncertainties. Providing definitive answers to such uncertain content may mislead patients, yet current assessment scales fail to evaluate this critical limitation [57,58]. Further research is needed to develop specific tools to enable more robust evaluation of LLM output quality.

### Conclusions

Our analysis found that GPT-4 provided high-quality responses to real-world questions related to scars and keloids, suggesting its potential as a useful patient education tool in scar and keloid treatment. The GPT-4 outputs were generally reliable and accurate but need improvement, primarily in readability and hallucinations.

## Supplementary material

10.2196/78838 Multimedia Appendix 1 Patient Education Materials Assessment Tool for Artificial Intelligence for evaluating the understandability (8 items) and actionability (3 items) of artificial intelligence–generated patient education text.

10.2196/78838 Multimedia Appendix 2 DISCERN-AI tool (7 core items) for assessing artificial intelligence–generated treatment information quality, with 1-3 scoring for each item (relevance, source clarity, date transparency, balance, additional support, uncertainty acknowledgment, and overall quality).

10.2196/78838 Multimedia Appendix 3 Natural Language Assessment Tool for Artificial Intelligence assessment framework: 5 domains (accuracy, safety, appropriateness, actionability, and effectiveness).

10.2196/78838 Multimedia Appendix 4 Reference Evaluation for AI assessment criteria: 3-item tool for validating artificial intelligence–generated references (real or fabricated, content support, and authoritative source status) on large language model reference hallucinations.

10.2196/78838 Multimedia Appendix 5 Supplementary tables for health care website–derived scar or keloid questions: includes 38 unique questions from 3 medical websites (Table S1), intraclass correlation coefficient values for assessment tools (Table S2), and artificial intelligence output evaluation scores (Patient Education Materials Assessment Tool for Artificial Intelligence, DISCERN-AI, Global Quality Scale, Natural Language Assessment Tool for Artificial Intelligence, readability, and reference quality) for website questions (Tables S3-S6).

10.2196/78838 Multimedia Appendix 6 Supplementary tables for Reddit-derived scar or keloid questions: includes subcategory-specific artificial intelligence output evaluation scores for all 16 question groups (Patient Education Materials Assessment Tool for Artificial Intelligence, DISCERN-AI, or Global Quality Scale: Table S1; Natural Language Assessment Tool for Artificial Intelligence: Table S2; and readability metrics: Table S3) and subcategory-specific reference evaluation results (Reference Evaluation for AI) for 3250 total cited references (Table S4), plus overall aggregate scores for all assessments.

## Abbreviations

AI: artificial intelligence
GQS: Global Quality Scale
ICC: intraclass correlation coefficient
LLM: large language model
NLAT-AI: Natural Language Assessment Tool for Artificial Intelligence
PEMAT: Patient Education Materials Assessment Tool
SMOG: Simple Measure of Gobbledygook
