# Supplementary material for: Evaluating GPT-4 Responses on Scars or Keloids for Patient Education: Large Language Model Evaluation Study
Source: JMIR Med Inform. 2026 Feb 27;14:e78838. doi: 10.2196/78838 (PMC12954683; doi:10.2196/78838)
Supplement: Multimedia Appendix 1 [file medinform-v14-e78838-s001.docx]

The PEMAT-AI tool is adapted from the Patient Education Materials Assessment Tool for Printable Materials[1].

**DOMAIN: UNDERSTANDABILITY**

1. The material makes its purpose completely evident.

0 - Disagree

1 - Agree

1. The material does not include information or content that distracts from its purpose.

0 - Disagree

1 - Agree

1. The material uses common, everyday language. Medical terms are used only to familiarize the audience with the terms. When used, medical terms are defined.

0 - Disagree

1 - Agree

1. The material uses the active voice.

0 - Disagree

1 - Agree

1. The material breaks or “chunks” information into short sections.

0 - Disagree

1 - Agree

1. The material uses visual cues (e.g., arrows, boxes, bullets, bold, larger font, highlighting) to draw attention to key points.

0 - Disagree

1 - Agree

1. The material presents information in a logical sequence.

0 - Disagree

1 – Agree

1. Numbers appearing in the material are clear and easy to understand. (score only if material includes numbers)

0 - Disagree

1 – Agree

No score – Not Applicable

**DOMAIN: ACTIONABILITY**

Item 1: The material clearly identifies at least one action the user can take (P and A/V)

0 - Disagree

1 - Agree

Item 2: The material addresses the user directly when describing actions (P and A/V)

0 - Disagree

1 - Agree

Item 3: The material breaks down any action into manageable, explicit steps (P and A/V)

0 - Disagree

1 - Agree

1. Shoemaker SJ, Wolf MS, Brach C. Development of the Patient Education Materials Assessment Tool (PEMAT): a new measure of understandability and actionability for print and audiovisual patient information. Patient education and counseling. 2014;96(3):395-403. [doi: 10.1016/j.pec.2014.05.027] [Medline: 24973195]
