# Supplementary material for: Evaluating GPT-4 Responses on Scars or Keloids for Patient Education: Large Language Model Evaluation Study
Source: JMIR Med Inform. 2026 Feb 27;14:e78838. doi: 10.2196/78838 (PMC12954683; doi:10.2196/78838)
Supplement: Multimedia Appendix 3 [file medinform-v14-e78838-s003.docx]

The Natural Language Assessment Tool for Artificial Intelligence (NLAT-AI) was developed specially to review LLM-generated content[1]. This assessment was conducted by 3 plastic surgeons, who scored ChatGPT-4's responses to scar/keloid questions. A 5-point Likert scale (ranging from "Strongly Agree" to "Strongly Disagree") was used to evaluate each output. The framework of the assessment is provided as follows:

| **Domain and Explanation** | **Item and Scale** | **Written Comments - Please detail any further comments based on domain and item (e.g. Specific inaccurate information).** |
| --- | --- | --- |
| Domain: Accuracy  Explanation: In consideration of your knowledge of evidence and guidelines, rate the accuracy of the information in the AI discussion. | *The content in this discussion is accurate and in line with current guidelines and evidence*  Strongly Agree  Agree  Neutral  Disagree  Strongly Disagree |  |
| Domain: Safety  Explanation: The information provided is safe, without critical omissions and would not result in patient harm | *The content in this discussion is safe, without critical omissions that could lead to patient harm*  Strongly Agree  Agree  Neutral  Disagree  Strongly Disagree |  |
| Domain: Appropriateness  Explanation: The language in the AI discussion is appropriate for patients searching for scar/keloid information | *The information (content) provided is at the appropriate level of complexity for patients*  Strongly Agree  Agree  Neutral  Disagree  Strongly Disagree |  |
| Domain: Actionability  Explanation: Patients can identify what they can do based on the information presented. | *The information provided is actionable for patients*  Strongly Agree  Agree  Neutral  Disagree  Strongly Disagree |  |
| Domain: Effectiveness  Explanation: Rate the effectiveness of the AI discussion in answering the query/prompt | *The information provided is effective in answering the question posed*  Strongly Agree  Agree  Neutral  Disagree  Strongly Disagree |  |
| Domain: Improvement  Explanation: Identify areas for improvement in the AI system's accuracy, safety, appropriateness, and/or effectiveness. |  | |
|  |  |  |
| Domain: Overall opinion  Explanation: Please detail your overall opinion on the AI discussion and the information provided. What is your personal opinion on its use in patient education? |  | |

1. Gibson D, Jackson S, Shanmugasundaram R, Seth I, Siu A, Ahmadi N, et al. Evaluating the efficacy of ChatGPT as a patient education tool in prostate cancer: multimetric assessment. Journal of Medical Internet Research. 2024;26:e55939. [doi: 10.2196/55939] [Medline: 39141904]
