# Supplementary material for: Evaluating GPT-4 Responses on Scars or Keloids for Patient Education: Large Language Model Evaluation Study
Source: JMIR Med Inform. 2026 Feb 27;14:e78838. doi: 10.2196/78838 (PMC12954683; doi:10.2196/78838)
Supplement: Multimedia Appendix 4 [file medinform-v14-e78838-s004.docx]

Reference Evaluation for AI (REF-AI), was developed to analyze references provided in ChatGPT-generated contents[1].

1. Are the references real?
   - Yes - The references cited in the ChatGPT section exist and correlate with real-world references.
   - No - The references cited in the ChatGPT section do not exist or do not correlate with real-world references.
2. Do the reference support the AI response?
   - Yes - The output of ChatGPT4 has very high correlation with the references cited in the ChatGPT section, with no inconsistencies or gaps.
   - No - The output of ChatGPT4 has little or no correlation with the reference cited in the ChatGPT section.
3. What are the sources of the references?
   - Yes - Direct reference to scientific research article, guidelines of government or healthcare organisation.
   - No - Not a source as per above.

1. Alkaissi H, McFarlane SI. Artificial Hallucinations in ChatGPT: Implications in Scientific Writing. Cureus. 2023;15(2):e35179. [doi: 10.7759/cureus.35179] [Medline: 36811129]
