# Supplementary material for: Evaluating GPT-4 Responses on Scars or Keloids for Patient Education: Large Language Model Evaluation Study
Source: JMIR Med Inform. 2026 Feb 27;14:e78838. doi: 10.2196/78838 (PMC12954683; doi:10.2196/78838)
Supplement: Multimedia Appendix 5 [file medinform-v14-e78838-s005.docx]

sTable 1. Questions on scar/keloid from healthcare websites.

| num | | question | url |
| --- | --- | --- | --- |
| 1 | What is a keloid scar? | | <https://my.clevelandclinic.org/health/diseases/keloid-scar> |
| 2 | Where do keloid scars grow? | | <https://my.clevelandclinic.org/health/diseases/keloid-scar> |
| 3 | What are the symptoms of a keloid scar? | | <https://my.clevelandclinic.org/health/diseases/keloid-scar> |
| 4 | What does a keloid look like? | | <https://my.clevelandclinic.org/health/diseases/keloid-scar> |
| 5 | Do keloid scars hurt? | | <https://my.clevelandclinic.org/health/diseases/keloid-scar> |
| 6 | What causes keloid scars? | | <https://my.clevelandclinic.org/health/diseases/keloid-scar> |
| 7 | How do you get keloids? | | <https://my.clevelandclinic.org/health/diseases/keloid-scar> |
| 8 | What are the risk factors for a keloid scar? | | <https://my.clevelandclinic.org/health/diseases/keloid-scar> |
| 9 | What are the complications of a keloid scar? | | <https://my.clevelandclinic.org/health/diseases/keloid-scar> |
| 10 | How is a keloid scar diagnosed? | | <https://my.clevelandclinic.org/health/diseases/keloid-scar> |
| 11 | How is a keloid scar treated? | | <https://my.clevelandclinic.org/health/diseases/keloid-scar> |
| 12 | Are there side effects of the treatment? | | <https://my.clevelandclinic.org/health/diseases/keloid-scar> |
| 13 | What can I expect if I have a keloid scar? | | <https://my.clevelandclinic.org/health/diseases/keloid-scar> |
| 14 | Do keloid scars go away? | | <https://my.clevelandclinic.org/health/diseases/keloid-scar> |
| 15 | How can I prevent keloid scars? | | <https://my.clevelandclinic.org/health/diseases/keloid-scar> |
| 16 | When should I see a healthcare provider? | | <https://my.clevelandclinic.org/health/diseases/keloid-scar> |
| 17 | What type of treatment do you recommend? | | <https://my.clevelandclinic.org/health/diseases/keloid-scar> |
| 18 | Are there side effects of treatment? | | <https://my.clevelandclinic.org/health/diseases/keloid-scar> |
| 19 | What caused the scar? | | <https://my.clevelandclinic.org/health/diseases/keloid-scar> |
| 20 | How do I prevent future injuries that lead to scars? | | <https://my.clevelandclinic.org/health/diseases/keloid-scar> |
| 21 | Is it safe for me to get piercings or tattoos? | | <https://my.clevelandclinic.org/health/diseases/keloid-scar> |
| 22 | What’s the difference between hypertrophic scars and keloid scars? | | https://sensushealthcare.com/frequently-asked-questions-keloid-scars/ |
| 23 | Are keloid scars contagious? | | https://sensushealthcare.com/frequently-asked-questions-keloid-scars/ |
| 24 | How do keloid scars form? | | https://sensushealthcare.com/frequently-asked-questions-keloid-scars/ |
| 25 | Are keloids dangerous? | | https://www.theprivateclinic.co.uk/treatments/dermatology-and-conditions/keloid-scars/faqs/ |
| 26 | Can keloids come back after treatment? | | https://www.theprivateclinic.co.uk/treatments/dermatology-and-conditions/keloid-scars/faqs/ |
| 27 | What is the difference between Keloid and Hypertrophic scars? | | https://www.theprivateclinic.co.uk/treatments/dermatology-and-conditions/keloid-scars/faqs/ |
| 28 | Can Piercings Cause Keloid Scars? | | https://www.theprivateclinic.co.uk/treatments/dermatology-and-conditions/keloid-scars/faqs/ |
| 29 | Can Tattoos Cause Keloid Scars? | | https://www.theprivateclinic.co.uk/treatments/dermatology-and-conditions/keloid-scars/faqs/ |
| 30 | What does keloid scar treatment involve? | | https://www.theprivateclinic.co.uk/treatments/dermatology-and-conditions/keloid-scars/faqs/ |
| 31 | How long does keloid scar treatment take? | | https://www.theprivateclinic.co.uk/treatments/dermatology-and-conditions/keloid-scars/faqs/ |
| 32 | How much does Keloid Scar treatment cost? | | https://www.theprivateclinic.co.uk/treatments/dermatology-and-conditions/keloid-scars/faqs/ |
| 33 | How soon will I see the results after keloid treatment? | | https://www.theprivateclinic.co.uk/treatments/dermatology-and-conditions/keloid-scars/faqs/ |
| 34 | How long will keloid treatment results last? | | https://www.theprivateclinic.co.uk/treatments/dermatology-and-conditions/keloid-scars/faqs/ |
| 35 | What can be done to prevent the development of keloid scars? | | https://www.finetouchdermatology.com/keloid-faq/ |
| 36 | How do steroids work in the treatment of keloid scars? | | https://www.finetouchdermatology.com/keloid-faq/ |
| 37 | How do lasers work to treat keloids? | | https://www.finetouchdermatology.com/keloid-faq/ |
| 38 | Are keloid scars dangerous or indicative of a larger health threat? | | https://www.finetouchdermatology.com/keloid-faq/ |

sTable 2. ICC^a^ for PEMAT-AI^b^, DISCERN-AI, GQS^c^ , and NLAT-AI^d^ on questions from Reddit.

| Assessment tools | ICC |
| --- | --- |
| PEMAT-AI | 0.73 |
| DISCERN-AI | 0.69 |
| GQS | 0.78 |
| NLAT-AI | 0.76 |

ICC^a^: Intraclass correlation coefficient; PEMAT-AI^b^: Patient Education Materials Assessment Tool for Artificial Intelligence; GQS^c^: Global Quality Scale; NLAT-AI^d^: Natural Language Assessment Tool for Artificial Intelligence.

sTable 3. PEMAT-AI^a^, DISCERN-AI, and GQS^b^ mean(SD^c^) score of ChatGPT-4’s output on questions from healthcare websites.

| Number of questions | PEMAT-AI | DISCERN-AI | GQS |
| --- | --- | --- | --- |
| 38 | 7.58±1.25 | 26.47±3.54 | 4.39±0.71 |

PEMAT-AI^a^: Patient Education Materials Assessment Tool for Artificial Intelligence; GQS^b^: Global Quality Scale; SD^c^: Standard Derivation.

sTable 4. NLAT-AI^a^ mean(SD^b^) score by ChatGPT question output on questions from healthcare websites.

| Number of questions | accuracy | safety | appropriateness | actionability | effectiveness |
| --- | --- | --- | --- | --- | --- |
| 38 | 4±0.9 | 4.4±0.7 | 4.3±0.8 | 4.1±0.7 | 4.1±0.7 |

NLAT-AI^a^: Natural Language Assessment Tool for Artificial Intelligence; SD^b^: Standard Derivation.

sTable 5. Mean(SD^a^) score of readability assessment by ChatGPT question output on questions from healthcare websites.

| Number of questions | Flesch Reading Ease score | Gunning Fog Index | Flesch-Kincaid Grade Level | The Coleman-Liau Index | SMOG^b^ Index |
| --- | --- | --- | --- | --- | --- |
| 38 | 49.16±6.9 | 12.48±2.45 | 12.41±2.36 | 12.9±2.36 | 10.95±2.26 |

SD^a^: Standard Derivation; SMOG^b^: Simple Measure of Gobbledygook.

sTable 6. Reference Evaluation Results of ChatGPT-4 Responses on Scars/Keloids on questions from healthcare websites.

| Number of questions | Reference number | Real References | Supporting  References | Authoritative References |
| --- | --- | --- | --- | --- |
| 38 | 298 | 288 | 283 | 260 |
