# Supplementary material for: Evaluating GPT-4 Responses on Scars or Keloids for Patient Education: Large Language Model Evaluation Study
Source: JMIR Med Inform. 2026 Feb 27;14:e78838. doi: 10.2196/78838 (PMC12954683; doi:10.2196/78838)
Supplement: Multimedia Appendix 6 [file medinform-v14-e78838-s006.docx]

sTable 1. PEMAT-AI^a^, DISCERN-AI, and GQS^b^ mean(SD^c^) score by ChatGPT question output.

| Questions | PEMAT-AI | DISCERN-AI | GQS |
| --- | --- | --- | --- |
| Questions on other respects | 80.4%(11.4**%)** | 25.9(3.2) | 4.3(0.8) |
| Questions on other treatments for scars or keloids | 62.5%(5.0**%)** | 26.5(1) | 3.5(0.6) |
| Questions on common treatments for scars or keloids | 71.1%(11.4**%)** | 27.2(3.4) | 4.4(0.8) |
| Questions on trauma-related scars/keloids | 76.2%(12.6**%)** | 26.5(3.5) | 4.1(0.8) |
| Questions on psychological issues caused by scars/keloids | 72.2%(10.9**%)** | 25.1(2.8) | 4.3(0.9) |
| Questions on at-home scar/keloid care | 73.3%(15.3**%)** | 25.3(4.5) | 4.3(0.6) |
| Questions on preoperative scar/keloid consultation | 71.9%(11.3**%)** | 26.4(3.2) | 4.3(0.8) |
| Questions on postoperative scar/keloid consultation | 80.5%(11.3**%)** | 26.7(3.4) | 4.2(0.8) |
| Questions on selection of treatments for scars or keloids | 76.2%(12.7**%)** | 26.1(3.3) | 4.3(0.8) |
| Questions on impact of scars/keloids on daily life | 80.0%(14.1**%)** | 26.5(4.9) | 4.5(0.7) |
| Questions on scar/keloid symptoms | 75.7%(12.7**%)** | 27.7(3.3) | 4(0.8) |
| Questions on scar camouflage | 66.7%(8.2**%)** | 25.2(2.8) | 4.3(0.8) |
| Questions on the impact of nutrition on scars/keloids | 73.3%(15.3**%)** | 26.7(5.1) | 4.3(0.6) |
| Questions on choosing doctors for scar/keloid treatment or related costs | 74.7%(11.4**%)** | 25.2(3.9) | 4.3(0.8) |
| Questions on old scars | 74.3%(13.4**%)** | 25.1(3.1) | 4.6(0.8) |
| Questions on scar/keloid prevention | 79.2%(13.1**%)** | 27.5(3.2) | 4.1(0.7) |
| Overall | 75.5%(12.2**%)** | 26.3(3.4) | 4.3(0.8) |

PEMAT-AI^a^: Patient Education Materials Assessment Tool for Artificial Intelligence; GQS^b^: Global Quality Scale; SD^c^: Standard Derivation.

sTable 2. NLAT-AI^a^ mean(SD^b^) score by ChatGPT question output.

|  | accuracy | safety | appropriateness | actionability | effectiveness |
| --- | --- | --- | --- | --- | --- |
| Questions on other respects | 3.8(0.8) | 4.2(0.7) | 4.4(0.8) | 4.1(0.7) | 4(0.7) |
| Questions on other treatments for scars or keloids | 4(0.8) | 3.8(1) | 4.3(1) | 4(0.8) | 3.5(0.6) |
| Questions on common treatments for scars or keloids | 3.7(0.8) | 4.2(0.8) | 4.5(0.6) | 4.1(0.6) | 4(0.8) |
| Questions on trauma-related scars/keloids | 3.9(0.8) | 4.2(0.8) | 4.6(0.6) | 3.9(0.8) | 4.3(0.7) |
| Questions on psychological issues caused by scars/keloids | 4.6(0.9) | 4.2(0.8) | 4(0.7) | 4(0.5) | 4.1(0.6) |
| Questions on at-home scar/keloid care | 4.3(1.2) | 4.3(0.6) | 4.3(0.6) | 3.7(0.6) | 4.3(0.6) |
| Questions on preoperative scar/keloid consultation | 3.8(0.8) | 4.3(0.8) | 4.1(0.7) | 4.2(0.7) | 4.2(0.7) |
| Questions on postoperative scar/keloid consultation | 3.8(0.8) | 4.3(0.7) | 4.3(0.8) | 4.2(0.6) | 4.1(0.8) |
| Questions on selection of treatments for scars or keloids | 4.1(0.8) | 4.5(0.7) | 4.3(0.7) | 4(0.7) | 4(0.8) |
| Questions on impact of scars/keloids on daily life | 4(1.4) | 5(0) | 5(0) | 3.5(0.7) | 4(0) |
| Questions on scar/keloid symptoms | 4.3(0.8) | 4.4(0.5) | 4.3(0.8) | 3.9(0.9) | 3.6(0.8) |
| Questions on scar camouflage | 4.5(0.8) | 4.2(0.8) | 4.2(1) | 4.2(0.8) | 3.8(0.8) |
| Questions on the impact of nutrition on scars/keloids | 3(0) | 4.7(0.6) | 5(0) | 4.3(0.6) | 4(1) |
| Questions on choosing doctors for scar/keloid treatment or related costs | 3.8(0.8) | 4.3(0.7) | 4.2(0.8) | 4(0.6) | 4.1(0.7) |
| Questions on old scars | 3.8(0.9) | 4.2(0.8) | 4.6(0.6) | 4(0.6) | 4(0.7) |
| Questions on scar/keloid prevention | 4(0.7) | 4.3(0.8) | 4.3(0.8) | 4.3(0.6) | 4(0.7) |
| Overall | 3.9(0.7) | 4.3(0.8) | 4.4(0.5) | 4.1(0.8) | 4.1(0.8) |

NLAT-AI^a^: Natural Language Assessment Tool for Artificial Intelligence; SD^b^: Standard Derivation.

sTable 3. Mean(SD^a^) score of readability assessment by ChatGPT question output.

|  | Flesch Reading Ease score | Gunning Fog Index | Flesch-Kincaid Grade Level | The Coleman-Liau Index | SMOG^b^ Index |
| --- | --- | --- | --- | --- | --- |
| Questions on other respects | 45.2(6.5) | 13.8(2.5) | 11.9(2.1) | 12.3(2.3) | 11.1(2.5) |
| Questions on other treatments for scars or keloids | 43.9(5.5) | 14.2(0.5) | 12.2(2) | 12.9(1.3) | 11.4(3.9) |
| Questions on common treatments for scars or keloids | 47.9(8.8) | 13.5(3.3) | 11.5(2.6) | 12.2(2.7) | 10.9(2.5) |
| Questions on trauma-related scars/keloids | 56.6(5.2) | 11.1(3.2) | 8(1.8) | 8.7(1.9) | 8.4(3.9) |
| Questions on psychological issues caused by scars/keloids | 44(7.7) | 14(2.3) | 12(2.8) | 12.4(1.9) | 11.2(2) |
| Questions on at-home scar/keloid care | 41.4(4.7) | 14.6(2.9) | 13.4(0.4) | 14.3(0.8) | 12.4(4.3) |
| Questions on preoperative scar/keloid consultation | 50.6(7.5) | 12.4(3.1) | 11.2(2.1) | 11.5(2.1) | 10.4(2.8) |
| Questions on postoperative scar/keloid consultation | 48.8(7.2) | 12.5(3.8) | 11.4(2.4) | 11.5(2.2) | 10.6(3.4) |
| Questions on selection of treatments for scars or keloids | 55.6(6.6) | 11.2(3.1) | 8.9(2.2) | 9.7(2.7) | 9.3(3.5) |
| Questions on impact of scars/keloids on daily life | 45.6(8) | 13.5(3) | 11.7(1.9) | 12.3(0.1) | 11(2.3) |
| Questions on scar/keloid symptoms | 36.9(3.6) | 16.5(1.6) | 14.4(0.6) | 14.3(1.3) | 13.8(1.5) |
| Questions on scar camouflage | 43.6(8.5) | 14.3(1.7) | 12.3(2.7) | 13.1(2.9) | 11.5(1.8) |
| Questions on the impact of nutrition on scars/keloids | 43.2(2.3) | 14.5(1.6) | 13.2(2.1) | 13.5(1.8) | 11.7(1.3) |
| Questions on choosing doctors for scar/keloid treatment or related costs | 51.6(6.1) | 11.9(2.3) | 9.4(2.3) | 9.8(2) | 9.6(2.5) |
| Questions on old scars | 50.9(5.1) | 11.9(3.4) | 11.1(2.3) | 11.4(1.8) | 9.7(3.4) |
| Questions on scar/keloid prevention | 48(7.6) | 13(3.7) | 11.5(2.3) | 11.6(1.9) | 10.8(3) |
| Overall | 50.1(8.1) | 12.7(3.3) | 12.4(2.5) | 12.8(2.6) | 11.3(3.2) |

SD^a^: Standard Derivation; SMOG^b^: Simple Measure of Gobbledygook.

sTable 4. Reference Evaluation Results of ChatGPT-4 Responses on Scars/Keloids.

| SUB | Reference number | Real References | Supporting  References | Authoritative References |
| --- | --- | --- | --- | --- |
| Questions on other respects | 248 | 214 | 207 | 197 |
| Questions on other treatments for scars or keloids | 41 | 32 | 30 | 32 |
| Questions on common treatments for scars or keloids | 426 | 376 | 362 | 357 |
| Questions on trauma-related scars/keloids | 143 | 132 | 127 | 126 |
| Questions on psychological issues caused by scars/keloids | 84 | 76 | 71 | 72 |
| Questions on at-home scar/keloid care | 29 | 22 | 20 | 22 |
| Questions on preoperative scar/keloid consultation | 362 | 326 | 311 | 309 |
| Questions on postoperative scar/keloid consultation | 503 | 432 | 416 | 416 |
| Questions on selection of treatments for scars or keloids | 712 | 631 | 600 | 597 |
| Questions on impact of scars/keloids on daily life | 17 | 17 | 16 | 16 |
| Questions on scar/keloid symptoms | 67 | 58 | 56 | 55 |
| Questions on scar camouflage | 52 | 43 | 41 | 40 |
| Questions on the impact of nutrition on scars/keloids | 27 | 26 | 26 | 26 |
| Questions on choosing doctors for scar/keloid treatment or related costs | 293 | 259 | 250 | 247 |
| Questions on old scars | 136 | 124 | 118 | 121 |
| Questions on scar/keloid prevention | 110 | 99 | 95 | 91 |
| Overall | 3250 | 2867 | 2746 | 2724 |
